# Neuropeptide Y mediates cardiac hypertrophy through microRNA-216b/FoxO4 signaling pathway

**DOI:** 10.7150/ijms.51133

**Published:** 2021-01-01

**Authors:** Jinghao Wang, Dan Hao, Lingfeng Zeng, Qianhui Zhang, Wei Huang

**Affiliations:** 1 Department of Pharmacy, the First Affiliated Hospital, Jinan University, Guangzhou 510630, China.; 2 Department of Cardiology, the First Hospital of Harbin, Harbin 150010, China.; 3 Department of Pharmacology, Harbin Medical University-Daqing, Daqing, Heilongjiang 163319, China.

**Keywords:** Cardiac hypertrophy, Neuropeptide Y, NPY1R, miR-216b, FoxO4

## Abstract

Cardiac hypertrophy (CH) is a major risk factor for heart failure accompanied by maladaptive cardiac remodeling. The role and potential mechanism of neuropeptide Y (NPY) in CH are still unclear. We will explore the role and the mechanism of NPY inactivation (NPY-I) in CH caused by pressure overload. Abdominal aortic constriction (AAC) was used to induce CH model in rats. NPY or angiotensin II (Ang II) was used to trigger CH model *in vitro* in neonatal rat ventricular myocytes (NRVMs). We found that NPY was increased in the heart and plasma of hypertrophic rats. However, Ang II did not increase NPY expression in cardiomyocytes. NPY-I attenuated CH as decreasing CH-related markers (ANP, BNP and β-MHC mRNA) level, reducing cell surface area, and restoring cardiac function. NPY inactivation increased miR-216b and decreased FoxO4 expression in CH heart. Moreover, NPY decreased miR-216b and increased FoxO4 expression in NRVMs which were reversed by NPY type 1 receptor (NPY1R) antagonist BIBO3304. MiR-216b mimic and FoxO4 siRNA (small interfering RNA) inhibited NPY/Ang II-induced myocardial hypertrophy* in vitro*. Meanwhile, BIBO3304 reversed the pro-hypertrophy effect of NPY* in vitro*. Collectively, NPY deficiency attenuated CH by NPY1R-miR-216b-FoxO4 axis. These findings suggested that NPY would be a potential therapeutic target for the prevention and treatment of cardiac hypertrophy.

## Introduction

Pathological cardiac hypertrophy is usually induced by pressure overload and continuous β-adrenergic receptor (β-AR)-activation, such as hypertension, ischemia, myocarditis and so on. Although the initial hypertrophic phase can indeed maintain cardiac output, continued pathological hypertrophy can destroy its beneficial effects and eventually lead to malignant arrhythmias, heart failure and sudden death 1-3. Understanding the key molecular events that mediate pathological hypertrophy is critical to developing new therapeutic strategies to prevent morbidity and mortality associated with myocardial hypertrophy.

Neuropeptide Y (NPY) is a 36 amino acid sympathetic neurotransmitter. It is known to be widely distributed in mammals' central and peripheral nervous systems 4, 5. It is involved in a variety of physiological activities including food intake, stress, cardiovascular and immune function, etc. 6-8. NPY is the most abundant neuropeptide in the heart and was first discovered in intramural sympathetic nerves that are closely connected to muscle cells and coronary arteries 9. It is also found in endocardial cells, myocardial cells, cardiac ganglia and parasympathetic nerve cells 8,10. Clinical studies have shown elevated plasma NPY levels in pathological conditions of sympathetic neuropathy, such as hypertension, left ventricular hypertrophy (LVH), myocardial infarction, and heart failure 8,11,12. It has been observed that the role of NPY in the occurrence and development of LVH is closely related to elevated plasma NPY levels during hypertension, while elevated plasma NPY levels are closely related to the severity of LVH 13, 14. Plasma NPY is considered a potential biomarker for diagnosis of CH and myocardial infarction (MI) 15, 16. Some studies have shown that providing exogenous NPY can induce cardiac hypertrophy in cultured primary cardiomyocytes and rats 17, 18. However, the role of NPY deficiency in the regulation of CH and its potential molecular mechanisms remain to be studied.

In the current study, we used abdominal aorta constriction rat models and NPY/Ang II-induced cardiomyocytes hypertrophy models to investigate whether NPY deficiency has protective effects on CH and myocardial dysfunction. Our findings suggest that NPY deficiency may provide new treatments for CH.

## Materials and methods

### Animal experiments

NPY inactivation rats were generously provided by Prof. Weidong Yong (Institute of Experimental Animal Science, Chinese Academy of Medical Sciences & Peking Union Medical College, Beijing, China). The experimental procedures involving animals in this study were approved by the Animal Ethics Committee of Harbin Medical University and the study was carried out in accordance with the Guide for the Care and Use of Laboratory Animals published by the US National Institutes of Health (NIH Publication, 8th Edition, 2011).The generation of NPY inactivation rat has been shown in previous studies 19. NPY-I rats were created using Zinc finger nuclease technology on an inbred alcohol-nonpreferring background. Body weights were decreased with no significant change in food intake in NPY-I rats. This study used male NPY-I (220 ± 20 g) and its wild-type (WT) Wistar rats. As described in previous studies, to establish a model of cardiac pressure overload, the rats underwent abdominal aorta constriction (AAC) surgery for 4 weeks 20. Rats were anaesthetized with ketamine-xylazine (100 mg/kg, 5 mg/kg, i.p.), and the abdominal aorta was exposed through a midline abdominal incision. Sham-operated rats went through the same process without aorta banding. The rats were randomly divided into the following four experimental groups (six rats in each group): Sham operation (Sham) group, NPY inactivation (NPY-I), Cardiac hypertrophy (CH) group, NPY inactivation treated AAC operation ((NPY-I+CH) group.

### Immunocytochemistry

Immunochemical analysis was performed to detect the expression of NPY in cardiac tissue. In short, the heart was fixed with 4% paraformaldehyde (pH 7.4) for 48 h. Heart tissue sections (5 μm thick) were incubated with 3% hydrogen peroxide in methanol to remove the endogenous peroxidase activity and then treated with normal serum to block non-specific binding. Subsequently, the sections were incubated with anti-NPY primary antibody (rabbit, 1:200, ab10980, Abcam, Cambridge, United Kingdom) at 4°C overnight, and secondary antibody (anti-rabbit,1:200, sc-2018, Santa Cruz, CA,USA) was incubated for 1 h. The sections were observed with diaminobenzidine (DAB) and counterstained with haematoxylin.

### Echocardiography and Histological analysis

Echocardiography was performed on the rats 4 weeks after CH under anesthesia. Echocardiography was performed using a Vevo2100 high-resolution imaging system (VisualSonics, Toronto, Ontario, Canada) with a 10 MHz imaging linear scanning probe transducer with M-mode recording. Left ventricular ejection fraction (EF), shortened fraction (FS), LV end-diastolic diameter (LVEDd), LV end-diastolic diameter (LVESd) dimensions, Left ventricular posterior wall depth (LVPWs) and left ventricular anterior wall thickness (LVAWs) were measured.

For histological analysis, the heart was fixed with 4% paraformaldehyde (pH 7.4) for 48 h. The fixed heart tissue was cut into 5 μm thick sections. Hematoxylin and eosin (H & E) staining was performed to detect or assess myocardial hypertrophy. Sections were imaged using a bright-field microscope (IX71, Olympus, Tokyo, Japan) at 200x magnification. Each observer randomly checks at least five (to eight) independent images from different (non-overlapping) regions.

### Neonatal rat ventricular myocytes culture and transfection

The procedure for culturing neonatal rat ventricular myocytes (NRVMs) is the same as previously described 21. NRVMs were grown in Dulbecco's modified Eagle's medium (DMEM; Hyclone, Logan, UT, USA) supplemented with 10% fetal bovine serum (FBS, Hyclone) under a humidified atmosphere of 95% air and 5% CO_2_ at 37°C. To induce hypertrophy, the cells were serum starved in DMEM for 12 h and then treated with NPY (100 nmol/L, Sigma-Aldrich, St. Louis, Missouri, USA) for 24 h 17.

MiR-216b mimic (50 nM), the negative control miRNA (miR-NC, 50 nM) and siRNA NC (50 nM) were synthesized by Guangzhou Ribo Bio Co., Ltd. The sequence of miR-216b mimic is 5'- GTCGTATCCAGTGCAGGGTCCGAGGTGCACTGGATACGACTCACATTT-3′, miR-NC is 5′-UUUGUACUACACAAAAGUACUG-3′ and siRNA NC is 5′-UUCUCCGAACGUGUCACGUTT-3′. FoxO4 siRNA (100 nM) was purchased from Santa Cruz Biotechnology, USA. NRVMs (1×10^5^ per well) were starved for 24 h in serum-free medium and then transfected with X-treme GENE siRNA transfection reagent (Roche, Germany) according to the manufacturer's instructions. 24 h after transfection, NRVMs were treated with NPY for 24 h or with 100 nmol/L Ang II (Sigma, St. Louis, MO, USA) for 48 h. Prior to processing NPY or Ang II, NPY1-R antagonist BIBO3304 and NPY5-R antagonist S25585 (1μM, Santa Cruz Biotechnology, USA) were pre-incubated for 12 h. BIBO3304 and antagonist S25585 diluted in ethanol and stored in dark and at 4°C before used.

### Cell surface area measurement

NRVMs were fixed with 4% paraformaldehyde for 0.5 h. The cell membrane was then infiltrated with 0.4% Triton X-100 for 1 h and blocked with goat serum for 1 h. Thereafter, the cells were incubated with anti-sarcomeric actin antibody (1:500, #19245,Cell Signaling Technology, Danvers, MA, USA) at 4°C overnight, and then conjugated with Cy3 goat anti-mouse antibody (1:1000, AP124, Sigma, St. Louis, MO, USA) for 1 h. Immunofluorescence was analyzed under a fluorescence microscope (Nikon, 80i, Japan). Cell surface area was quantified and averaged by measuring 60 random cells from three experiments with Image-Pro Plus Data Analysis Software.

### Quantitative real-time reverse transcription-PCR (qRT-PCR)

Total RNA was extracted from NRVMs in different treatments or cardiac tissues using TRIZOL reagent (USA, Invitrogen, USA) according to the manufacturer's protocol. SYBR Green was used to determine the levels of NPY, ANP, BNP, β-MHC, FoxO4 (with GAPDH as internal control) and miR-216b mRNA (with U6 as internal control) on the Roche LightCycler^®^480 Real Time PCR system (Roche, USA). The sequences of primers were presented in Table 1, and miR-216b-5p RT: 5'-GTCGTATCCAGTGCAGGGTCCGAGGTGCACTGGATACGACTCACATTT-3'; U6 RT: 5'-CGCTTCACGAATTTGCGTGTCAT-3'. The amount of target (2^-ΔΔCT^) was obtained by normalizing with respect to endogenous reference and with respect to the calibrator (average of the control samples).

### Luciferase assay

To generate a reporter vector bearing miRNA-binding site, the 3-untranslated region (3′-UTR) of FoxO4 was synthesized by Sangon (Shanghai, China). The construct was inserted into multiple cloning sites downstream of the luciferase gene (SacI and HindIII sites) in the pMIR-REPORT luciferase miRNA expression reporter vector (Ambion, USA). To test the binding specifcity, the sequences that interacted with the seed sequence of miR-216b-5p were mutated (from AGAGAUU to UAUAUAA for the miR-216b-5p binding site), and the mutant FoxO4 3'-UTR was inserted into an equivalent luciferase reporter plasmid. For the luciferase assay, 0.1 μg of luciferase reporters containing 3′-UTR were co-mingled with miR-216b-5p mimic or miR-216b-5p inhibitor or miR-NC into HEK-293 cells using lipofectamine 2000 (Invitrogen, USA). As an internal control, a 10 ng of renilla luciferase reporters was also included. After 48 h transfection, the cells were collected and double luciferase activity was measured by luminometer according to the manufacturer's instructions (Promega Corporation).

### Western blot

As described in previous studies, total protein was extracted from the NRVMs and heart tissue 21. Briefly, proteins were separated by electrophoresis on an SDS-polyacrylamide gel and transferred wet to a nitrocellulose filter. Membranes were incubated with anti-FoxO4 (1:100, sc-373877, mouse monoclonal, Santa Cruz, USA) antibody overnight at 4°C with the following secondary antibodies for 1 h at room temperature in the dark. The images were captured by the Odyssey CLx Infrared Imaging System (LI-COR Biosciences, Lincoln, NE, USA). Anti-β-actin (1:1000, sc-8432, mouse polyclonal, Santa Cruz, CA, USA) antibody is internal control.

### Detection of NPY levels

NPY levels were measured using an ELISA kit according to the manufacturer's instructions (H167, Nanjing Jiancheng Bioengineering Institute, Nanjing, China).

### Data analysis

The data are presented as the mean ± SD. The statistical analyses were used by one-way or two-way ANOVA (for groups of ≥ 3) and t-test (for 2 groups). In all cases, *P < 0.05* was considered to be statistically significant. The data were analyzed using GraphPad Prism 5.0.

## Results

### NPY level was elevated in CH

**Figure 1:**
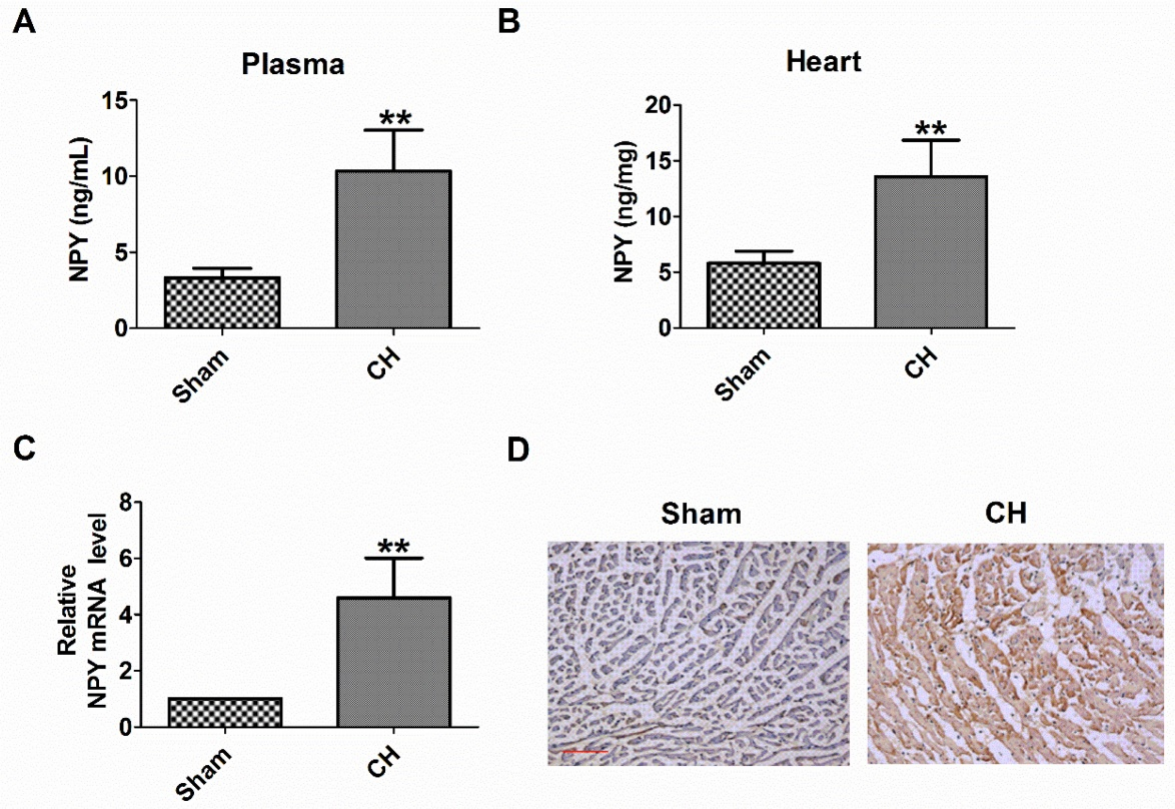
** Levels of NPY are increased in the plasma and heart of rat with CH.** (A) NPY level in the plasma by ELISA (n = 6). (B) NPY level in the heart by ELISA (n = 6). (C) The relative mRNA level of NPY (n = 6). (D) Immunocytochemistry analysis of NPY was examined in CH myocardium (200X). Scale bar = 50 μm. Brown stain represented positive signal (n = 4). **P < 0.01 vs Sham.

First, we examined the plasma and heart NPY level in CH rats. We found that the levels of NPY protein in the plasma and heart of CH rats were significantly higher than those in the Sham group (Fig 1A, B). Consistently, NPY mRNA expression and NPY expression determined by qRT-PCR and immunochemical analysis, respectively, also increased in the hearts of CH rats (Fig 1C, D). Therefore, these results indicated an increase in NPY levels in CH *in vivo*.

### NPY inactivation attenuates cardiac hypertrophy in vivo

**Figure 2:**
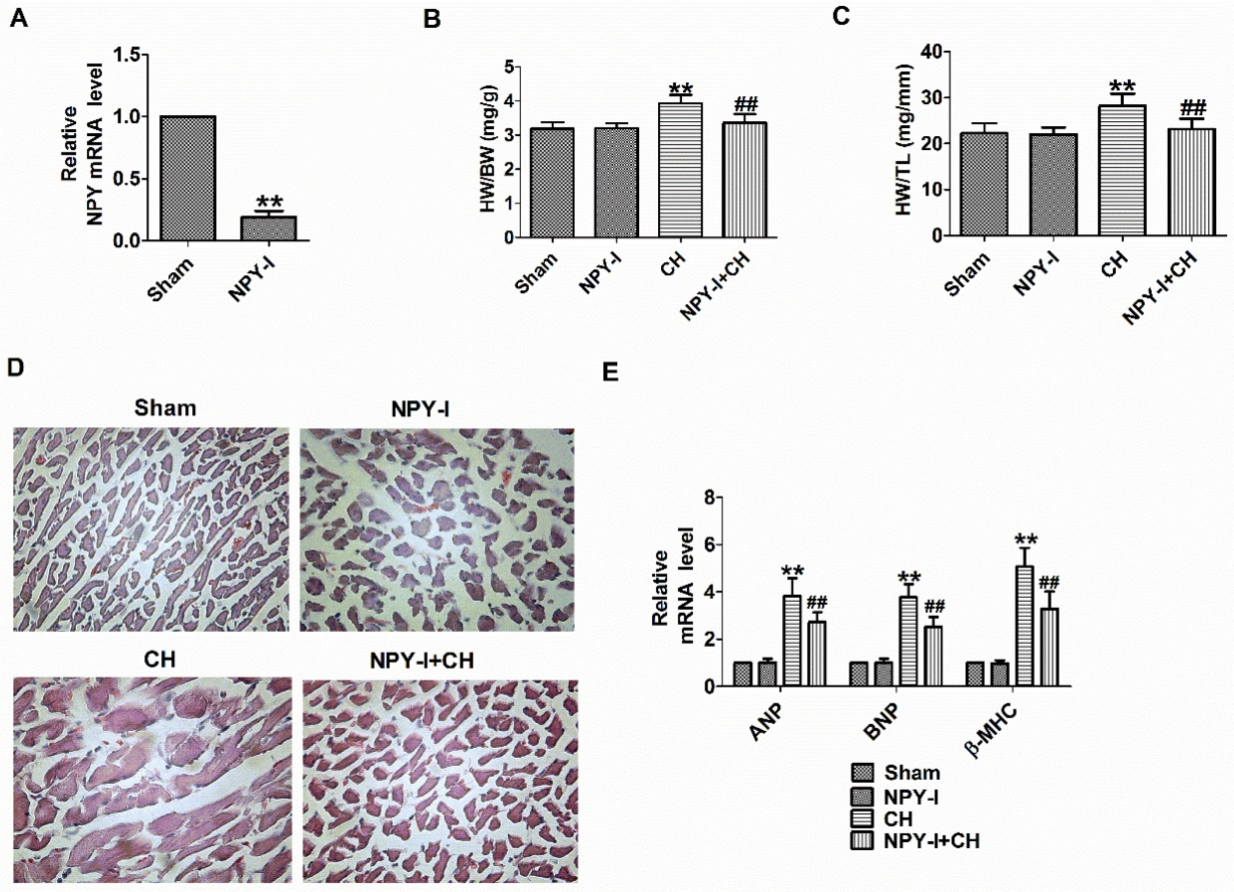
** Effect of NPY inactivation in cardiac hypertrophy in vivo.** (A) The relative mRNA level of NPY (n = 4). (B) The statistical results of the heart weight (HW)/body weight (BW) ratios (n = 6). (C) The statistical results of the HW/tibia length (TL) ratios (n = 6). (D) Representative histological results of the H&E staining of the rat heart tissues (n = 4, 400X). (E) The relative mRNA levels of the hypertrophic markers ANP, BNP, and β-MHC (n = 6). **P < 0.01 vs Sham; ^##^*P* < 0.01 vs CH.

First, NPY expression was detected in heart tissues of NPY-I rats. Compared with the sham operation group, NPY mRNA levels in NPY-I rats were significantly reduced (Fig. 2A). Later we found that NPY-I significantly reduced HW/BW (heart weight to body weight) and HW/TL (HW/tibia length) ratios in CH rats (Fig. 2B, C). In addition, an enlargement of the cardiomyocytes was found in CH rats by H&E, while NPY-I significantly reduced the surface area of ​​cardiomyocytes in CH rats (Fig. 2D). At the same time, mRNA levels of ANP, BNP, and β-MHC increased significantly in CH compared to the sham group, while the increase of hypertrophic markers in CH were suppressed by NYP-I (Fig. 2E). It is worth noting that compared with the sham operation group, HW/BW, HW/TL, cell area, and the mRNA levels for the hypertrophic biomarker (ANP, BNP, and β-MHC) in NPY-I rats were all no obvious abnormalities (Fig. 2B-E).

**Figure 3:**
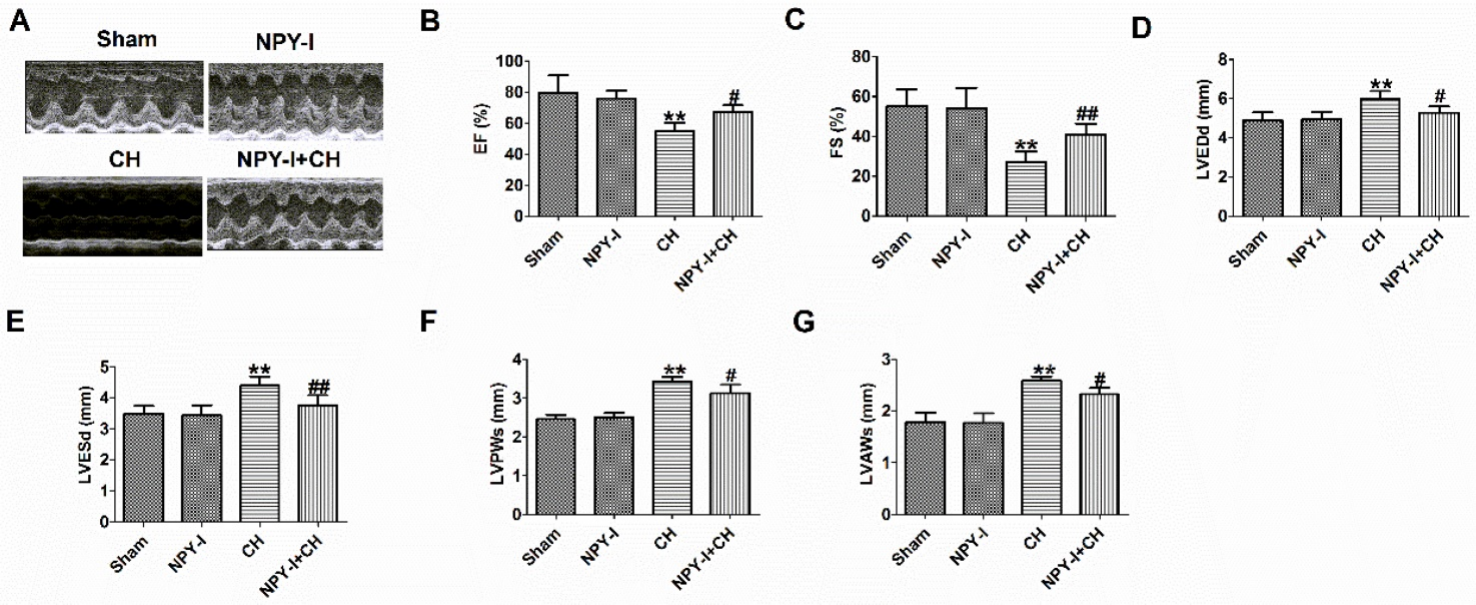
** Effect of NPY inactivation on cardiac function in cardiac hypertrophy.** (A) Representative M-mode images of heart function. (B) Ejection fractions (EF). (C) Fractional shortening (FS). (D) LV end-diastolic diameter (LVEDd). (E) LV end-systolic diameter (LVESd). (F) LV posterior wall depth (LVPWs). (G) LV anterior wall thickness (LVAWs).n = 6. **P < 0.01 vs Sham; ^#^*P* < 0.05, ^##^*P* < 0.01 vs CH.

In addition, echocardiography showed a marked decrease in ejection fraction (EF) and fractional shortening (FS) of CH hearts, and an increase in LVEDd, LVESd, LVPWs and LVAWs, indicating impaired cardiac function (Fig. 3A-G). NPY-I reduced the deterioration of left ventricular function in CH rats (Fig. 3A-G). Interestingly, EF, FS, LVEDd, LVESd, LVPWs and LVAWs were all also no obvious abnormalities in NPY-I and sham rats (Fig.3 A-G). These data indicated that NPY-I had a protective effect on CH and significantly alleviated cardiac dysfunction during CH.

### NPY-NPY1R regulates miR-216b and FoxO4

**Figure 4:**
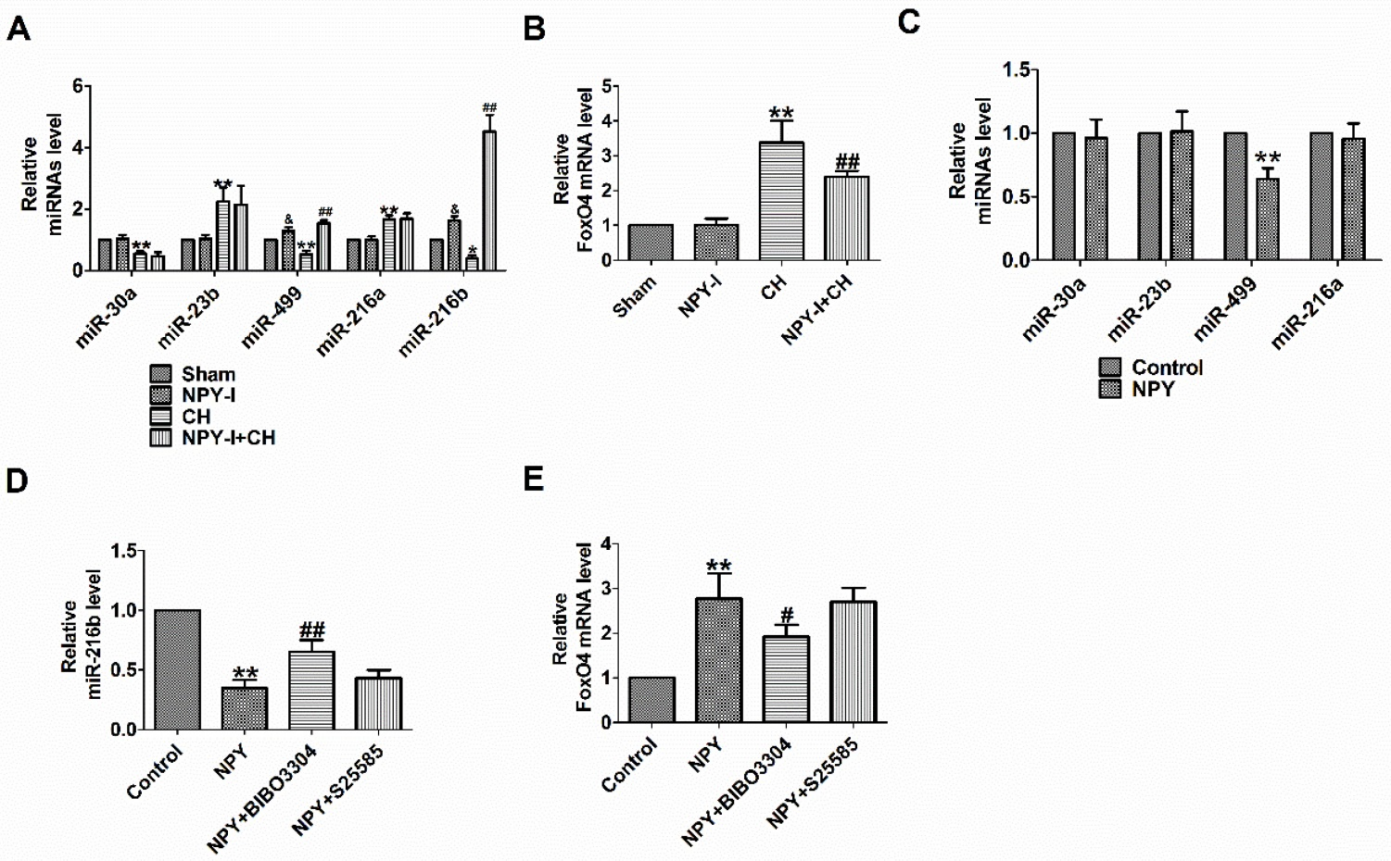
** NPY regulates miR-216a and FoxO4.** (A) The relative level of the miRNAs in CH. (B) The relative level of the FoxO4 in CH. (C) The relative level of the miRNAs in NPY treated cardiomyocytes. (D) The relative level of the miR-216b in NPY treated cardiomyocytes. (E) The relative level of the FoxO4 in NPY treated cardiomyocytes (n = 4). A&B:*P < 0.05, **P < 0.01 vs Sham; ^&^*P* < 0.05 vs Sham;^ #^*P* < 0.05, ^##^*P* < 0.01 vs CH. C-E: **P < 0.01 vs Control;^ #^*P*<0.05, ^##^*P* < 0.01 vs NPY.

FoxO4 is a member of the widely expressed fork head (Fox) transcription factor O family, which also includes FoxO1, O3, and O6. FoxO proteins regulate a variety of biological processes, including oxidative stress, metabolism, immunity and apoptosis 22. FoxO4 is likely to be the potential target gene of miR-216b predicted by Target Scan software. MiR-216b inhibited myocardial autophagy and apoptosis during myocardial infarction and hypoxia 23. MiR-216a accelerates proliferation and fibro-genesis via targeting PTEN and SMAD7 in human cardiac fibroblasts 24. Some studies showed that FoxO4 is the target gene of miR-23b or miR-499 25, 26. We previously found NPY regulated miR-499 in myocardial infarction rat model 26. One study demonstrated that NPY regulated miR-30a in an in vitro model of Alzheimer's disease 27. MiR-23b, miR-499 and miR-30a are involved in the progress of CH 28-30. In order to detect the relationship between NPY and miRNAs (miR-30a, miR-23b, miR-499, miR-216a and miR-216b) or FoxO4, we evaluated the expression of miRNAs and FoxO4 by quantitative real-time PCR. Our results showed that miR-30a, miR-499 and miR-216b decreased significantly more, and miR-23b or miR-216a increased in CH *in vivo*, while FoxO4 increased more, which was reversed by NPY-I, and NPY-I increased miR-216b and miR-499 expression in Sham rats (Fig. 4A, B). Moreover, the expression of miR-216b increased more than miR-499 in CH rats, so it was used for subsequent experiments (Fig. 4A). However, there was no significant abnormality in FoxO4 expression in NPY-I rats compared with the sham groups (Fig. 4 B). In addition, there were no significant abnormality in miRNAs (miR-30a, miR-23b, and miR-216a) expression in NPY treated cardiomyocytes compared with the control groups (Fig. 4 C). Moreover, the expression of miR-216b decreased more than miR-499 in NPY treated cardiomyocytes (Fig. 4C, D).

NPY causes positive contraction in cardiomyocytes hypertrophy through NPY1R, and it is a predominant receptor subtype in the heart 12, 31. Another research showed that NPY induced NRVMs hypertrophy via NPY1R 32*.* NPY5R has been reported to mediate NPY-induced cardiomyocytes hypertrophy 33. Therefore, to understand which NPY receptor (NPY1R and NPY5R) is involved in NPY/miR-216b axis, we identified the mRNA of miR-216b with NPY receptor (NPY1R and NPY5R) antagonist in NPY treated cardiomyocytes. We found that NPY significantly decreased miR-216b and increased FoxO4 expression in NRVMs, whereas NPY1R antagonist BIBO3304 significantly increased miR-216b and decreased FoxO4 expression in NPY treated cardiomyocytes (Fig. 4D, E). Interestingly, NPY5R antagonist S25585 without changing miR-216b and FoxO4 expression in NPY treated cardiomyocytes (Fig. 4D, E).

### Interplay between miR-216b and FoxO4

**Figure 5:**
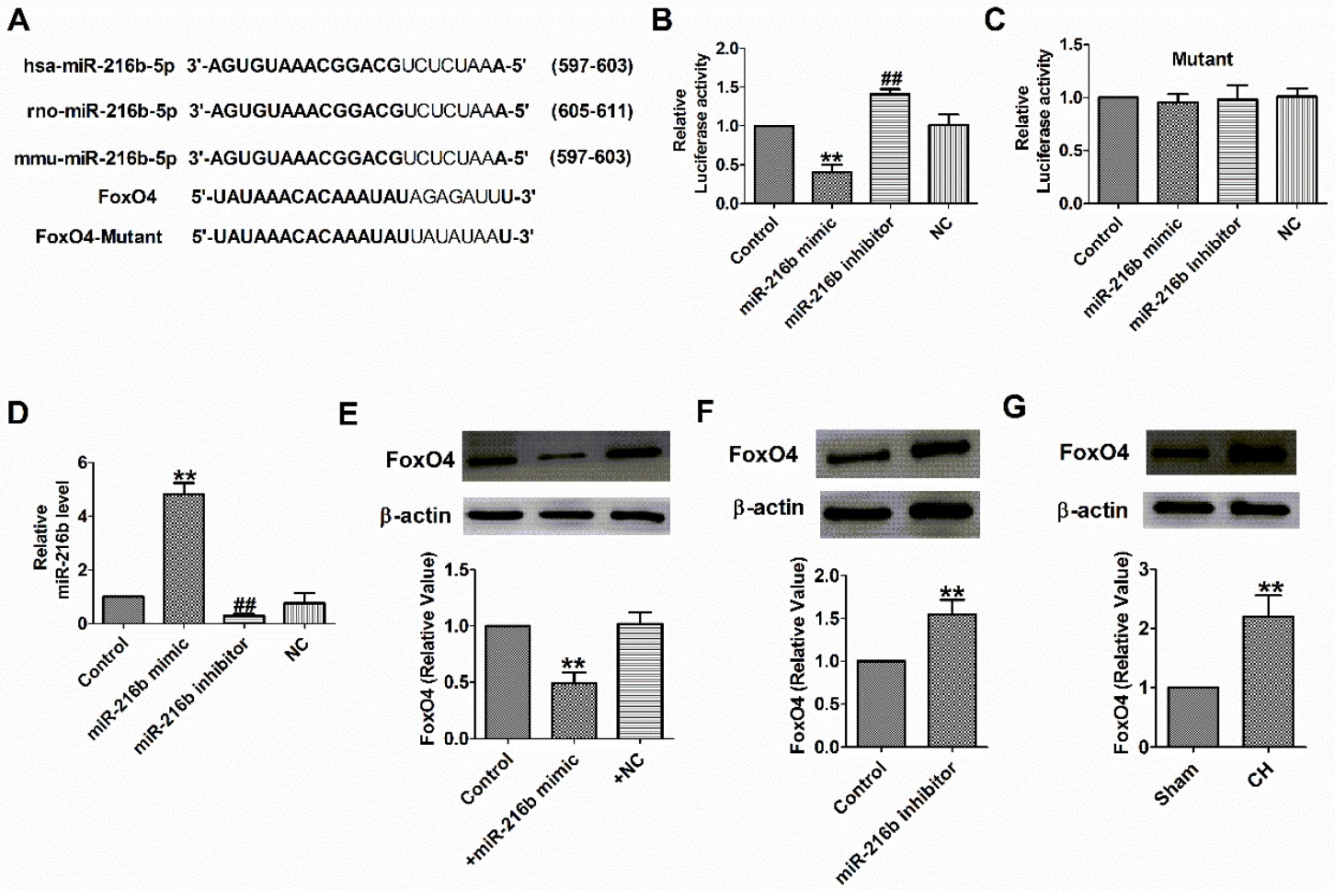
** FoxO4 was the direct target of miR-216b.** (A) Sequence alignment show between miR-216b and the binding sites in the 3'UTR of the FoxO4 gene. (B&C) The interaction between miR-216b and its binding sites in the 3'UTR of FoxO4 or mutated was examined by luciferase assay in HEK293 cells (n = 4). (D) The relative mRNA level of miR-216b in cardiomyocytes (n = 4). (E&F)The expressions of FoxO4 protein were measured in cardiomyocytes (n = 3). (G) The expressions of FoxO4 protein were measured in CH (n = 3). B-E: **P < 0.01, ^##^*P* < 0.01 vs Control. F: **P < 0.01 vs Sham.

Subsequently, we need to check whether miR-216b directly targets FoxO4. We used targetscan software to make predictions and found a conserved binding site for the miR-216b in the 3 binding site FoxO4 (Fig. 5A). Luciferase assay showed that miR-216b mimic significantly inhibited the luciferase activity of the constructed plasmid containing 3′-UTR of FoxO4, while miR-216b inhibitor showed the opposite effect, but not that of the mutant 3′-UTR (Fig. 5B,C). Next, we examined miR-216b level and the protein expression of FoxO4 after transfected with miR-216b mimic, miR-216b inhibitor or NC in NRVMs. We found that miR-216b level was significantly increased after miR-216b mimic transfection and decreased after miR-216b inhibitor transfection (Fig. 5D). MiR-216b mimic significantly inhibited FoxO4 protein expression while miR-216b inhibitor had an opposite effect (Fig. 5E, F), and verified the relationship between miR-216b and FoxO4. Finally, we found that the protein expression of FoxO4 increased in rat CH (Fig. 5G). These results revealed that FoxO4 was a direct target of miR-216b.

### NPY1R/miR-216b/FoxO4 pathway is critical for NPY induced cardiac hypertrophy

**Figure 6:**
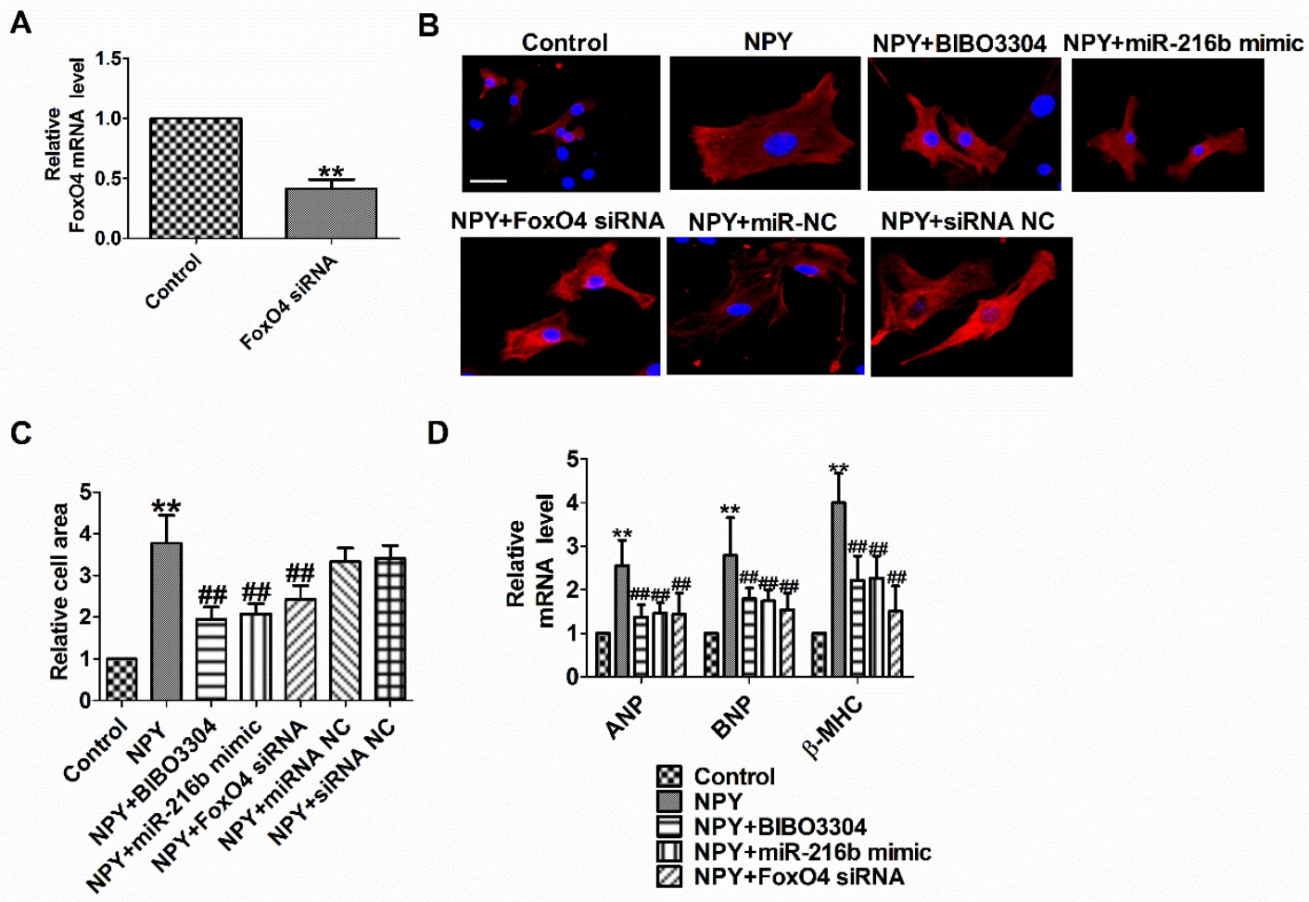
** NPY1R/miR-216b/FoxO4 pathway is critical for NPY induced cardiac hypertrophy.** (A) The relative mRNA level of FoxO4 (n = 4). (B&C) Microscopic images of the cardiomyocytes and statistical results of cell surface of cardiomyocytes. (n = 4 independent experiments, 100X). Scale bar = 20 μm. (D) The relative mRNA levels of the hypertrophic markers ANP, BNP, and β-MHC (n = 6). **P < 0.01 vs Control;^ ##^*P*<0.01 vs NPY.

To functionally analyze whether NPY1R/miR-216b/FoxO4 signal pathway is a necessary condition for NPY-induced cardiac hypertrophy, we transfected NPY into myocardial cells alone or in combination with BIBO3304 or miR-216b mimic or FoxO4 siRNA. Compared with the control group, FoxO4 mRNA level was significantly decreased in FoxO4 siRNA group (Fig. 6A). As expected, BIBO3304, miR-216b mimic and FoxO4 siRNA abated the pro-hypertrophy effects of NPY in cardiomyocytes, which was exhibited by an increase in cell surface area, ANP, BNP, and β-MHC mRNA (Fig. 6B-D). Taken together, our results indicated that NPY mediated CH* in vitro* through NPY1R/miR-216b/FoxO4 pathway.

### miR-216b and FoxO4 siRNA attenuates Ang II induced cardiac hypertrophy *in vitro*

**Figure 7:**
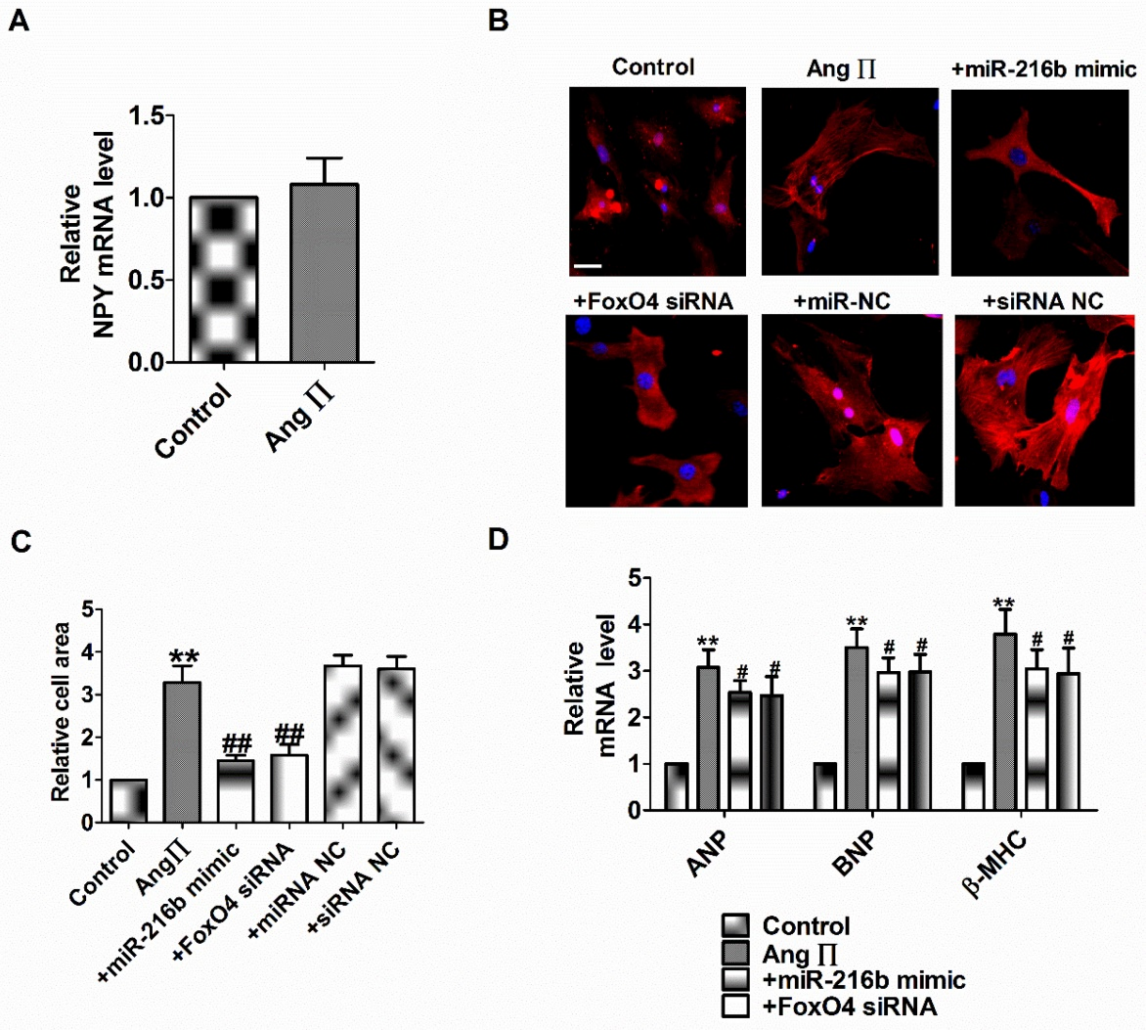
** miR-216b and FoxO4 siRNA attenuates Ang II induced cardiac hypertrophy *in vitro*.** (A) The relative mRNA level of NPY (n = 4). (B&C) Microscopic images of the cardiomyocytes and statistical results of cell surface of cardiomyocytes. (n = 4 independent experiments, 100X). Scale bar = 20 μm. (D) The relative mRNA levels of the hypertrophic markers ANP, BNP, and β-MHC (n = 6). **P < 0.01 vs Control;^ #^*P* < 0.05, ^##^*P* < 0.01 vs Ang II.

NPY1R/miR-216b/FoxO4 pathway is critical for NPY induced cardiac hypertrophy. Therefore, we explore whether the effect of NPY1R/miR-216b/FoxO4 same as NPY in Ang II induced cardiomyocytes hypertrophy. Firstly, we found Ang II did not induce NPY release in cardiomyocytes* in vitro* as in rat CH *in vivo* (Fig. 7A). Next, we transfected Ang II into myocardial cells alone or in combination with miR-216b mimic or FoxO4 siRNA to study its effect on cardiomyocytes hypertrophy induced by Ang II. We observed that Ang II increased cell surface area, ANP, BNP, and β-MHC mRNA level in cardiomyocytes (Fig. 7B-D). MiR-216b mimic and FoxO4 siRNA significantly decreased cell surface area, ANP, BNP, and β-MHC mRNA level in Ang II treated cardiomyocytes (Fig. 7B-D).

## Discussion

**Figure 8:**
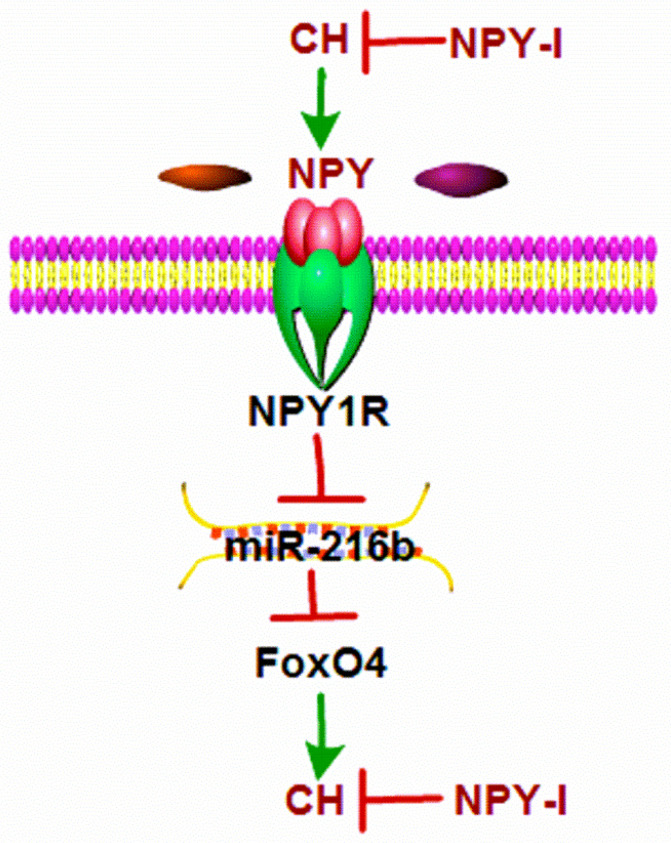
**Schematic diagram for the proposed neuropeptide Y mediated cardiac hypertrophy signaling pathways.** NPY was up-regulated in CH; NPY inactivation inhibited CH and improved cardiac function in CH rats; miR-216b mimic and FoxO4 siRNA attenuates cardiac hypertrophy in vitro and FoxO4 is a direct target of miR-216b; and NPY mediates CH in vitro through NPY1R/miR-216b/FoxO4 pathway.

The main finding of this study were: (1) NPY was up-regulated in CH; (2) NPY inactivation inhibited CH and improved cardiac function in CH rats; (3) miR-216b mimic and FoxO4 siRNA attenuated cardiac hypertrophy *in vitro* and FoxO4 is a direct target of miR-216b; (4) NPY mediated CH* in vitro* through NPY1R/miR-216b/FoxO4 pathway (Fig.8). These findings suggested that NPY deficiency would be beneficial for CH repair.

Previous studies have reported that NPY is associated with the progression of multiple diseases, such as hypertension, diabetes, CH and other cardiovascular diseases 12, 34-36. Circulating NPY levels in patients with cardiac hypertrophy and heart failure were higher than those in the control group 13, 36. Our findings are consistent with these previous results, and we demonstrate elevated ventricular and plasma NPY levels in CH rats. Long-term subcutaneous injection of NPY may cause cardiac dysfunction and cardiac hypertrophy in rats, and NPY treatment may also cause cardiomyocytes hypertrophy 17, 18. However, it is unclear whether NPY knockout exerts an anti-hypertrophy effect. In this study, we evaluated for the first time the role of NPY-I in AAC surgery induction rats. Our results showed that AAC induced significant CH, which was characterized by increased cell area in CH rats, increased expression of ANP, BNP, and β-MHC, and impaired cardiac function. NPY-I relieves all AAC-induced cardiac hypertrophy, confirming the cardio-protective property of NPY deficiency in CH.

FoxO4 activates Arg1 transcription in endothelial cells in response to MI, resulting in down-regulation of nitric oxide and up-regulation of neutrophil infiltration into the infarct area 37. FoxO4 knockdown inhibited ROS production and myocardial apoptosis, ultimately reducing the infarction area and improving cardiac dysfunction caused by myocardial ischemia-reperfusion injury 38. Our preliminary experimental results showed that FoxO4 siRNA significantly inhibited the apoptosis of H_2_O_2_-treated cardiomyocytes 26. MiRNAs are small endogenous non-protein coding RNA, which is about 22 nucleotides in length, and play a role in negative regulation of gene expression by pairing with the protein-coding genes mRNAs′ 3′ UTR region 39. MiRNAs are known to play important roles in cardiovascular diseases, including CH and heart failure 39, 40. Recent study showed that NPY induced cardiomyocytes hypertrophy via attenuating miR-29a-3p in neonatal rat cardiomyocytes 41. We found deletion of NPY reduced myocardial ischemia, improved cardiac function, and inhibited cardiomyocytes apoptosis by NPY type 1 receptor/miR-499/FoxO4 axis 26. In addition, FoxO4 is most likely a potential target gene for the miR-216b predicted by Target Scan software. Therefore, we tested whether inhibitory effect of NPY-I on cardiac hypertrophy is related to the miR-216b/FoxO4 pathway and inhibitory effect of miR-216b mimic and FoxO4 siRNA on cardiac hypertrophy *in vitro*. We found that NPY-I can increase the miR-216b and decreased the FoxO4 expression induced by AAC. At the same time, NPY reduced miR-216b and increased FoxO4 expression in cardiomyocytes. Next, FoxO4 as a direct target of miR-216b was validated by using a luciferase reporter gene assay and protein expression detections.

NPY is co-released with norepinephrine and stimulates vasoconstriction, vascular and cardiomyocytes hypertrophy through NPY1R, and which is a predominant receptor subtype in the heart 12, 42. NPY induced NRVMs hypertrophy via NPY1R 33. Our previously study showed that NPY-I protected the heart from dysfunction and attenuated ischemic induced apoptosis by NPY1R in MI 26. Therefore, we tested whether NPY regulated CH by NPY1R/miR-216b/FoxO4 pathway. As expected, we found that NPY1R antagonist BIBO3304 can reverse the decrease in miR-216b and the increase in FoxO4 in NPY treated cardiomyocytes. At the same time, BIBO3304, miR-216b mimic and FoxO4 siRNA can decrease cardiomyocytes cell area, ANP, BNP, and β-MHC expression in NPY induced CH *in vitro*. Together, NPY mediates CH through NPY1R/miR-216b/FoxO4 signaling pathway.

Previous studies showed that sympathetic stimulation induced overflow of NPY by angiotensin II in spontaneously hypertensive rats and is released simultaneously with norepinephrine 12, 43. In this study, we found Ang II did not increase NPY level in cultured cardiomyocytes; perhaps there are no sympathetic neurons. Next, we found miR-216b mimic and FoxO4 siRNA significantly decreased cell surface area, ANP, BNP, and β-MHC mRNA level in Ang II induced cardiomyocytes hypertrophy as NPY.

In summary, our present study revealed that NPY deficiency plays a crucial role in anti-hypertrophy in cellular and animal models. The benefit effect of NPY deficiency may be mediated by the NPY1R/miR-216b/FoxO4 signaling pathway. Therefore, the regulation of NPY deficiency may provide a new method for treating myocardial hypertrophy.

## Figures and Tables

**Table 1:** Primes used for qRT-PCR [sequences 5' - 3'].

Gene	Forward	Reverse
miR-216b-5p	AAATCTCTGCAGGCAA ATGTGA	ACATTTGCCTCCAGAGATTTTT
U6	GCTTCGGCACATATACTAAAAT	CGCTTCACGAATTTGCGTGTCAT
GAPDH	AAGAAGGTGGTGAAGCAGGC	TCCACCACCCAGTTGCTGTA
NPY	GGCCAGATACTACTCCGCTCTGCG	TTCACAGGATGAGATGAGATGTG
ANP	ACCTGCTAGACCACCTGGAG	CCTTGGCTGTTATCTTCGGTACCGG
BNP	GAGGTCACTCCTATCCTCTGG	GCCATTTCCTCCGACTTTTCTC
β-MHC	CCGAGTCCCAGGTCAACAA	CTTCACGGGCACCCTTGGA
FoxO4	CTTTCTGAAGACTGGCAGGAATGTG	GATCTAGGTCTATGATCGCGGCAG
